# Cardiovascular and metabolic consequences of obesity

**DOI:** 10.3389/fphys.2015.00032

**Published:** 2015-02-10

**Authors:** Geoffrey A. Head

**Affiliations:** Neuropharmacology Laboratory, Baker IDI Heart and Diabetes InstituteMelbourne, VIC, Australia

**Keywords:** obesity, hypertension, dyslipidaemia, cardiovascular risk, metabolic syndrome

## Introduction

The alarming increase in the incidence and impact of obesity in Australia requires urgent national focus at all levels of government and the community. The reason for the call to action has been due to the realization of the enormity of the health issues of being overweight or obese. There is clearly increased risk of metabolic disease, cardiovascular complications and premature death. The predictions outlined in a Victorian Government report suggest increases in the prevalence of being overweight or obese of between 0.4 and 0.8%, respectively per year. From the levels in 1995 in which 65% of adult males and 50% of adult females were classified as overweight or obese, we now expect levels to increase to 83% of adult males and 75% of females by 2025 (Haby and Marwick, 2008). Thus, being overweight or obese will become very much the “norm” for Australians unless this issue is addressed with appropriate evidence based allocation of resources for prevention, reduction and mitigation. Importantly, we must clearly understand and act on the short term and long term impact on the health and well-being of the individual of being overweight or obese. The early consequences of obesity involve a range of metabolic changes that have been grouped together to define the “metabolic syndrome” (Cameron et al., 2009a). These include dyslipidaemia and changes to metabolic homeostasis including insulin resistance which is the primary feature of type 2 diabetes. The progression from obesity to metabolic dysfunction to diabetes ultimately occurs unless there are significant interventions. However, the real danger lies in the long term effect of obesity related metabolic disease on cardiovascular risk associated with greater rates of atherosclerosis, hypertension, vascular disease and cardiovascular mortality. There is also a considerable alteration in the quality of life related to public stigma, reduced mobility, higher levels of unemployment and psychological sequelae including depression. Thus, the purpose of the current review is to bring together the latest research on the health complications of obesity as it becomes the dominant driver for the health sector and a dominant financial burden for the community.

## The metabolic consequences of obesity for health

There is very strong evidence for the causative association between obesity and metabolic disease including dyslipidaemia, insulin resistance and the development of type 2 diabetes although the mechanisms are not fully understood. The dyslipidaemia associated with obesity includes elevated triglycerides (Howard et al., 2003) higher levels of specific forms of low density lipoprotein (LDL) particles (Williams and Krauss, 1997) and reduced high density lipoprotein (HDL) levels and these changes are all known to be atherogenic (Howard et al., 2003). Being overweight incurs a 2 or more fold elevated risk of high triglycerides, reduced HDL and metabolic syndrome. Being obese adds a staggering 4 fold increased risk of diabetes (Cameron et al., 2009b). The 5 year follow up of the AusDiab population study in 2005 indicates that the incidence of impaired glucose tolerance, impaired fasting glucose or type 2 diabetes in Australia in 2005 was 14% (Williams et al., 2010). A more alarming statistic is that predicted future adult levels of diabetes (from 2010 to 2030) will increase by 69% in developing countries and by 20% in developed countries (Shaw et al., 2010). The latter rise parallels quite closely the rate of increase in obesity in the Australian community which is expected given their well-established association.

Indigenous Australians have a higher rate of diabetes as well as high rates of dyslipidaemia and metabolic syndrome. A recent population study in the Northern Territory shows that almost one-third of those aged over 35 and over half of those over 55 years have diabetes (O'Dea et al., 2008). Clearly there needs to be considerable extra effort in such groups if we are to “bridge the gaps.”

## Long term impact of obesity on health and well-being

### Hypertension and ischaemic heart disease

The cardiovascular consequences of obesity are arguably the most important due to the high risk that being overweight or obese incurs particularly in men. While 30–40% of the elevated triglyceride and reduced HDL are due to obesity in women, this is associated with only 13% of myocardial infarction. For the men the reverse is true. Only 16–17% of the lipid changes are due to obesity but this has been attributed to a 32% greater incidence of myocardial infarction (Cameron et al., 2009b).

The impact of obesity is broader than the common view that the associated dyslipidaemia is a major cause of coronary artery disease. The vast majority of new cases of hypertension are related to obesity (Garrison et al., 1987). The mechanism of the hypertension has been extensively studied particularly in animal models. Central actions of leptin in the hypothalamus result in increased sympathetic activity to peripheral blood vessels and particularly to the kidney causing elevated blood pressure (Head et al., 2014). Obesity characterized by increased fat and also lean body mass results in increased oxygen demand, increased blood volume, cardiac output, stroke volume, and eccentric cardiac hypertrophy (Hall et al., 2002). Together with concentric cardiac hypertrophy from elevated blood pressure, coronary artery disease leads to both systolic and diastolic dysfunction (Lawrence and Kopelman, 2004). Prolonged obesity and hypertension may also contribute to glomerular hypertension, hyper-filtration and loss of renal function and increased salt sensitivity (Hall et al., 2003). High circulating levels of leptin result in leptin resistance and loss of appetite suppression while the central pressor actions are amplified (Head et al., 2014).

While hypertension is usually asymptomatic, increased blood pressure is associated with increased risk of stroke, myocardial infarction and renal failure. A 20 year follow up of over 15000 subjects found that compared to normal subjects with BMI less than 25, obese subjects with BMI>30 had a 1.5–2.5 fold risk of coronary heart disease (death or hospitalization), heart failure, stroke, venous thromboembolism, and atrial fibrillation (Murphy et al., 2006).

### Sleep and activity disordered breathing

Breathlessness upon minor exercise is common in obesity due to the presence of abdominal and chest deposits of fat that hinder breathing by limiting the excursion of the diaphragm (Lawrence and Kopelman, 2004). However, the more serious impact upon respiration comes during recumbent sleep when increased fat and soft tissue in the throat obstruct the airway which is known as obstructive sleep apnoea (OSA) (Lawrence and Kopelman, 2004). This results in elevation of nocturnal blood pressure and is strongly linked to an increased risk of developing hypertension. Half of those with morbid obesity have OSA and half of the patients with OSA have hypertension (Silverberg et al., 1995). The mechanism appears to involve central activation of the chemoreflex and increased sympathetic activity (Narkiewicz and Somers, 1999). Consequences of OSA include daytime sleepiness, increased risk of traffic accidents, increased risk of stroke and pulmonary hypertension (Lawrence and Kopelman, 2004).

### Vascular disease

The complications associated with obesity and in particular when type 2 diabetes develops, include peripheral vascular disease, coronary artery disease and endothelial dysfunction. While it is difficult to separate the influence of obesity from the other risk factors, studies have shown that obesity is independently associated with endothelial dysfunction (Avogaro and de Kreutzenberg, 2005). Mechanisms may involve adipokines and cytokine induced oxidative stress and low level inflammation. Vascular markers of inflammation such as plasminogen activation inhibitor, fibrinogen and c-reactive protein are also positively correlated with obesity (Lawrence and Kopelman, 2004). Thus, it is not surprising that obesity and the ensuing dyslidipdaemia are associated with deep venous thrombosis, pulmonary embolism and atherosclerotic arterial disease (Lawrence and Kopelman, 2004).

### Gout, arthritis, and gall stones

Gout and hyperuricemia is a growing health problem particularly in affluent countries such as Australia where it is commonly associated with metabolic syndrome, type 2 diabetes, obesity and hypertension (Robinson et al., 2012). Obesity is strongly associated with gouty arthritis but also with osteoarthritis. More recently it has been realized that obesity is also associated with rheumatoid arthritis (Stavropoulos-Kalinoglou et al., 2011).

### Cancer

While the mechanisms relating obesity to cancer are unclear due to the confounding effects of metabolic changes, dyslipidaemias, growth factors such as insulin-like growth factor and changes to hormone metabolism, there is a definite association for specific cancers. A meta-analysis has estimated that obesity accounts for 5% of all cancers but particularly for cancer of the endometrium (39%), the kidney (25%), and gallbladder (25%) (Bergstrom et al., 2001).

### Complications in surgery

Due to the more likely presence of coronary artery disease and hypertension, the obese patient is at greater risk of numerous medical issues that can occur during and subsequent to surgery but which can be minimized with appropriate precautions and monitoring (Choban and Flancbaum, 1997). Obese surgical patients have increased risk of deep vein thrombosis and surgical site infection. There is also a well-known obesity paradox where there is better survival of the obese patient after myocardial infarct and coronary bypass surgery that is independent of age, sex, medical therapy, and other factors (Amundson et al., 2010).

## Managing the psychological and social impact of obesity

More and more there is realization of the impact of the stigma of obesity on individuals (O'Brien et al., 2013). This perhaps is surprising given the increasing prevalence of obesity. Anti-obesity discrimination can occur even among health professionals and obesity researchers. The causes are many but the perception of lack of personal control, gluttony, and laziness as well as the promotion of distortedly thin body image aspirations all play a part (O'Brien et al., 2013). A recent study demonstrated that the level of anti-fat prejudice along with other prejudicial attitudes manifested resulted in obesity discrimination. People with obesity are less likely to be successful at job interviews and once employed are assigned less desirable tasks and receive lower salaries than colleagues (O'Brien et al., 2013).

Obesity is often associated with a range of psychological disturbances including anxiety, depression, poor body image, binge eating disorders which are exacerbated by social stigma and chronic diseases such as arthritic pain and functional disability. Depression is highest in those with disordered eating or those with symptoms of bodily pain. Overall quality of life is severely reduced particularly in the morbidly obese patients (Dixon, 2010). While psychological impact and quality of life is a major problem for the individual and for society, it is a particularly difficult area to research the underlying mechanisms due to the many interacting factors.

The reality is that the maintenance of body weight is so strongly defended by inbuilt physiological processes that only an extremely small percentage of the population can sustain weight reduction in the long term. On the other hand, storage of excess calories occurs readily, surreptitiously and without immediate consequences such that during adulthood the weight gain appears almost inevitable and is not at all gluttony or lack of self-control. Obesity needs to be considered as a chronic medical disease state requiring a multi-step approach to weight management as suggested by Kushner (2014). Simply telling a patient to lose weight is not a long term strategy and considerable investment in understanding the driving factors including social issues, lifestyle, food marketing strategies and the like is necessary. At the same time there needs to be a much greater acceptance of the biological causes and investment in research to develop effective therapies in addition to those currently available (Kushner, 2014). While gastric surgical techniques have been shown to have long term weight reduction effectiveness, it is abundantly clear that this is not a solution for more than a small number of the huge percentage of the population that are affected (Kushner, 2014).

## Conclusions

The alarming increase in the proportion of the Australian community and particularly the indigenous community that are overweight, obese or morbidly obese must be considered as one of most pressing health issues of our time. The adverse changes in metabolic homeostasis, lipid metabolism, pro-thrombotic state and the development of diabetes coalesce to exponentially increase cardiovascular risk. Thus, obesity is a major contributor to cardiovascular disease, hypertension, myocardial infarcts and stroke. For the individual, the decreased mobility, discomfort, lower limb and joint pain, social standing, and mental health status result in a serious deterioration of quality of life particularly for those most affected. Dieting is obviously the most effective strategy for being overweight or obese but is difficult to maintain in the long-term without additional pharmacological or surgical interventions. It is important to also recognize that obesity is a disease state and that negative social attitudes particularly from health professionals may be driving patients away from seeking appropriate professional help.

### Conflict of interest statement

The author declares that the research was conducted in the absence of any commercial or financial relationships that could be construed as a potential conflict of interest.
